# Low-dose aspirin protects unexplained recurrent spontaneous abortion *via* downregulation of HMGB1 inflammation activation

**DOI:** 10.3389/fendo.2022.914030

**Published:** 2022-11-17

**Authors:** Xiaofeng Xu, Jing Wang, Damin Zhu, Jiaqian Yin, Jinxian Liu, Xiao Wu, Wenjuan Yang, Qian Hu, Yu Ren, Zhiguo Zhang, Ping Zhou, Zhaolian Wei, Huijuan Zou, Yunxia Cao

**Affiliations:** ^1^ Reproductive Medicine Center, Department of Obstetrics and Gynecology, the First Affiliated Hospital of Anhui Medical University, Hefei, China; ^2^ National Health Commission (NHC) Key Laboratory of Study on Abnormal Gametes and Reproductive Tract (Anhui Medical University), Hefei, China; ^3^ Key Laboratory of Population Health Across Life Cycle (Anhui Medical University), Ministry of Education of the People’s Republic of China, Hefei, Anhui, China; ^4^ Center for Reproductive Medicine, Ma’anshan Maternal and Child Health Hospital, Ma’anshan, Anhui, China; ^5^ Anhui Province Key Laboratory of Reproductive Health and Genetics (Anhui Medical University), Hefei, Anhui, China; ^6^ Biopreservation and Artificial Organs, Anhui Provincial Engineering Research Center, Anhui Medical University, Hefei, Anhui, China

**Keywords:** aspirin, high mobility group box protein 1, unexplained recurrent spontaneous abortion, inflammation, pregnancy

## Abstract

**Background:**

High mobility group box protein 1 (HMGB1) is considered as a kind of sterile inflammatory mediators, which is an overexpression in patients with unexplained recurrent spontaneous abortion (URSA). Specific targeting effect of aspirin on HMGB1 has been revealed. Our previous studies have explored the application of HMGB1 as a therapeutic target of aspirin in URSA disease of mice model and human, but the dynamic process of aspirin downregulating HMGB1 concentration has not been demonstrated.

**Methods:**

From December 2018 to November 2020, women with URSA (*n* = 91) and control women (*n* = 90) with no history of recurrent abortion or adverse pregnancy were included in the Reproductive Medicine Center of the First Affiliated Hospital of Anhui Medical University. ELISA was applied to detect the concentrations of HMGB1 and IFN-γ in the peripheral blood. Thirty-one URSA patients were monitored for low-dose aspirin treatment (2 and 4 weeks), the changes of HMGB1 and IFN-γ concentrations in peripheral blood of URSA patients before and after using aspirin were compared, and pregnancy outcomes after aspirin treatment were followed up.

**Results:**

The levels of HMGB1 in peripheral blood were significantly higher in URSA patients compared with controls, decreasing trends of HMGB1 and IFN-γ concentrations in plasma of URSA patients were observed after treatment with low-dose aspirin continuously, and the expression of HMGB1 was positively correlated with IFN-γ. There were no birth abnormalities in the babies of the URSA patients treated with aspirin.

**Conclusions:**

High levels of HMGB1 may be one of the pathogenesis of URSA. Low-dose aspirin may provide protective effect on the HMGB1-triggered URSA.

## Introduction

Child is the happiness source of a family, but 1–2% of couples suffer from recurrent spontaneous abortion (RSA); they underwent the loss of three or more consecutive pregnancies, approximately 40–50% of these recurrent miscarriages are unexplained, which is known as unexplained recurrent spontaneous abortion (URSA). Previous studies detected that URSA was related to immune factors. Maternal tolerance to the semi-allogeneic fetus in mammalian pregnancy requires significant immunological changes in its own system and maternal fetal interface (1). The initial Th1/Th2 paradigm proposed that Th1 cytokines (IL-2), IFN-γ, and TNF are harmful to the maintenance of pregnancy; Th2 cytokines have a role of downregulating the harmful cytokines (2). Successful pregnancy requires a fine immune balance established between mother and fetus, whereas the peace between the two organisms can sometimes be broken by untimely factors, such as inflammation. Sensing the presence of inflammation, then mounting an effective response is essential for every step of female reproduction.

High mobility group box protein 1 (HMGB1) was first discovered as a novel proinflammatory mediator in 1999 in studies of sepsis (3). It belongs to a family member of damage-associated molecular patterns (DAMPs), and has been proved to be involved in the progression and deterioration of many diseases, including trauma, autoimmune diseases, and cancer. For example, HMGB1 can downregulate Treg response whereas upregulate Th17 response to exacerbate liver injury and inflammation in patients with chronic hepatitis B (4). In addition, HMGB1 can promote the thrombotic process by participating in the prothrombotic cross-communication between platelets, monocytes, and neutrophil granulocytes (5). Upon released into the extracellular milieu from damaged or necrotic cells, HMGB1 becomes highly proinflammatory, recruiting immune cells and inducing them to produce proinflammatory cytokines (6). Its proinflammatory response is related to different receptors, namely, Toll-like receptor 2 (TLR2), TLR4, TLR9, and receptor for advanced glycation end products (RAGE). HMGB1 and its receptors are found to be overexpressed in diverse pathologies. Several researches have showed that the death of trophoblasts can release DAMPs that activates TLRs signaling (7, 8). Furthermore, HMGB1-induced signaling can activate NF-κB pathway, thereby promoting inflammation.

As an inflammatory driver, HMGB1 plays a very important role in the whole process of pregnancy. In early pregnancy, appropriate HMGB1 has been proved to be a key regulator of embryo implantation and development. In addition, labor initiation also needs to be driven by appropriate inflammatory factors. However, excessive HMGB1 levels in maternal circulation or intrauterine cavity will lead to pathological pregnancy. For example, patients suffered from intra-amniotic inflammation will deliver early when their amniotic fluid is affected by high concentrations of HMGB1 and IL6 (9), and injecting HMGB1 into pregnant mice can lead to premature delivery (10). Moreover, HMGB1 excessively elevated in peripheral blood and chorionic villi of URSA (11).

Given that HMGB1 can have devastating effect on the multiple inflammatory disorders, it has been identified as a biomarker and therapeutic target (12). For example, the administration of HMGB1 antagonists can significantly improve survival of sepsis (6). In recent years, it has been discovered that salicylic acid (SA) can bind to HMGB1. Notably, glycyrrhizin is a known HMGB1 inhibitor, SA overlaps its binding sites (12, 13). Aspirin is commonly known as acetylsalicylic acid, which can be rapidly deacetylated to SA by esterase in human plasma (14). Pharmacologically, as a typical non-steroidal anti-inflammatory drug, aspirin works by irreversibly inhibiting the enzymatic activities of cyclooxygenases 1 and 2 (COX1 and COX2). Of interest, HMGB1 induces the expression of the gene encoding COX2, and these low levels of SA suppress this induction (15). Aspirin is widely used to reduce the risk of heart disease, atherosclerosis, and some cancers. In addition, it is also applied for the aim of optimizing the chance of living birth in women undergoing assisted reproductive technology (16). Moreover, our previous studies were the first time to explore the application of HMGB1 as a therapeutic target of aspirin in URSA disease of mice model and human (17, 18).

In this study, we demonstrated the dynamic trend of aspirin downregulating HMGB1 and proved the correlation between HMGB1 and inflammation. We provided the evidence of the efficacy and security of aspirin in relieving recurrent miscarriage.

## Materials and methods

### Ethics declaration

This study was approved by the medical ethics committee of the First Affiliated Hospital of Anhui Medical University and the relevant hospitals (ethical approval no. PJ2018-02-09, clinical trial registration: ChiCTR1800015403).

### Subjects

**Figure 1:**
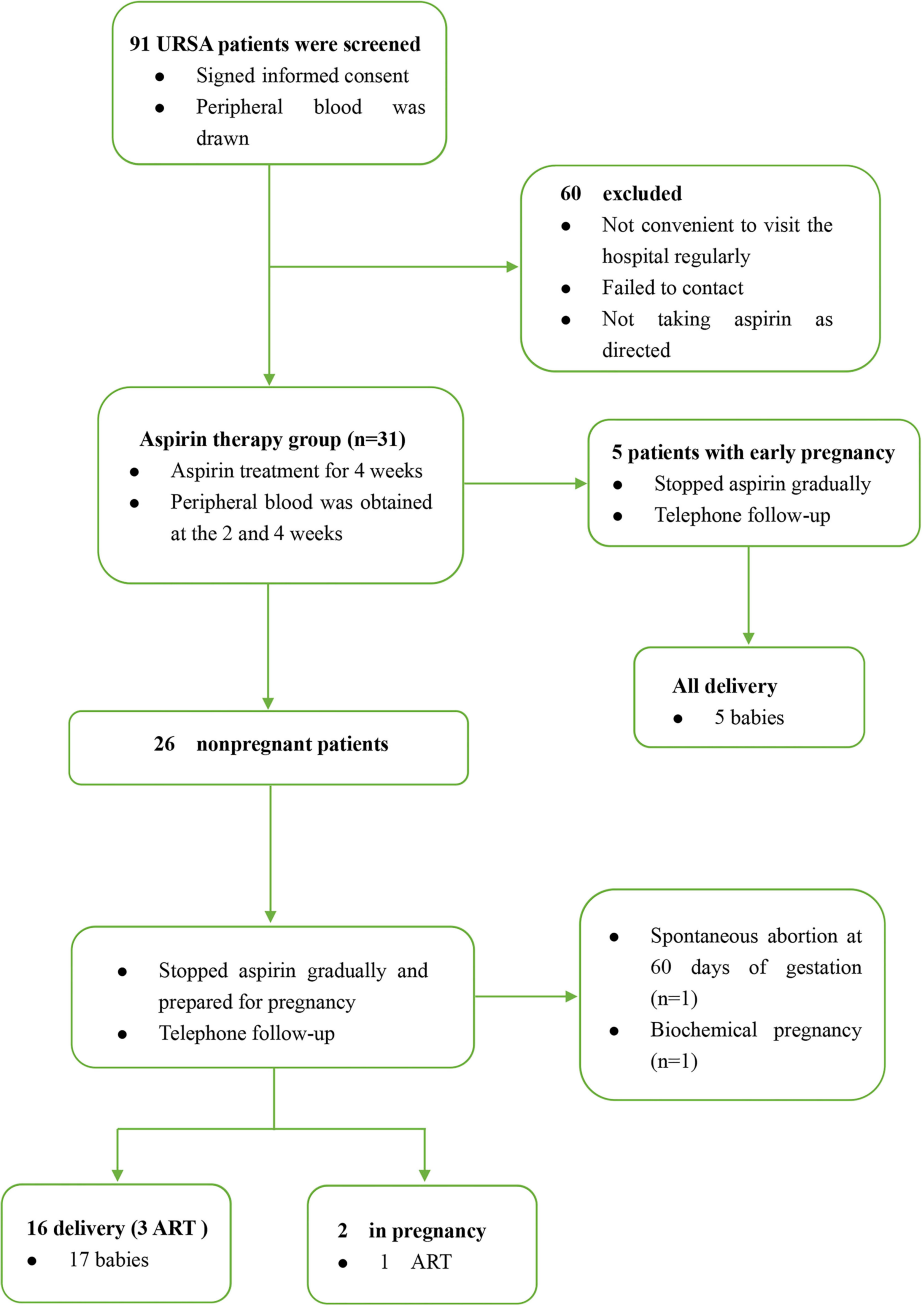
The sample collection and observation process of URSA group. ART, assisted reproductive technology.

From December 2018 to November 2020, we strictly screened 91 URSA patients from the Reproductive Medicine Center of the First Affiliated Hospital of Anhui Medical University. They did not receive aspirin treatment frequently before they were enrolled with a mean age of 29.7 ± 4.1 years. All participants cannot find a specific cause that resulted in RSA; the exclusion criteria were as follows: (1) infectious diseases such as toxoplasmosis, rubella, cytomegalovirus, and bacterial vaginosis; (2) abnormal genital tract structure such as septate uterus and bicornuate uterus; (3) abnormal endocrine tests such as sexual hormone and thyroid function; (4) abnormal autoantibody tests such as antinuclear antibody (ANA) and anticardiolipin antibody (ACL); (5) abnormal coagulation function; (6) genetic abnormalities such as fetal aneuploidy and patients or their partners with abnormal chromosome karyotype. They provided peripheral blood and signed informed consent at their first visit. Patients who are willing to enter the aspirin treatment group were prescribed with oral aspirin at a dose of 50 mg per day (100 mg per day if weight more than 65 kg); if there was abnormal bleeding, stop the medicine immediately. We collected venous blood of the patients at 2 and 4 weeks after taking aspirin. Finally, due to the influence of COVID-19, some patients failed to come to the hospital in time, and some patients did not take the medicine according to the dose; only 31 URSA patients completed the periodic monitoring of aspirin treatment, five of them were already in early pregnancy (4–6 weeks) at the time of inclusion. The patients stopped aspirin gradually after they completed the aspirin monitoring treatment and prepared for pregnancy under our guidance. The sample collection and observation process of URSA group is shown in **Figure 1**.

In addition, 90 nonpregnant healthy women who had not received aspirin treatment recently with a mean age of 28.6 ± 3.1 years were enrolled as the control group. None of them had a history of recurrent abortion, ectopic pregnancy, still birth, infection, autoimmune disease, and abnormal biochemical indexes.

The blood samples taken from URSA and control groups were centrifuged at 400*g* for 10 min; then, we collected the upper plasma. All specimens were stored in the −80°C refrigerator for future use.

### ELISA assays

The frozen plasma was restored to room temperature, and then the concentrations of HMGB1 and IFN-γ were detected using ELISA kits (USCN, SEA399Hu; USCN, SEA049Hu). All operations were performed according to the manufacturer’s directions. The absorbance was measured at 450 nm by Microplate Reader. We calculated the final concentration using the standard curve.

### Statistical analysis

Data were analyzed by Graphpad Prism software; figures were plotted with Graphpad Prism. All results were presented as mean ± standard error of the mean (SEM). The differences between two groups were evaluated using unpaired Student’s *t*-test. One-way ANOVA was used for multiple groups comparison. Pearson’s correlation analysis was used. *P* value < 0.05 was considered as significant.

## Results

### The upregulated HMGB1 plasma concentrations in URSA patients

**Figure 2:**
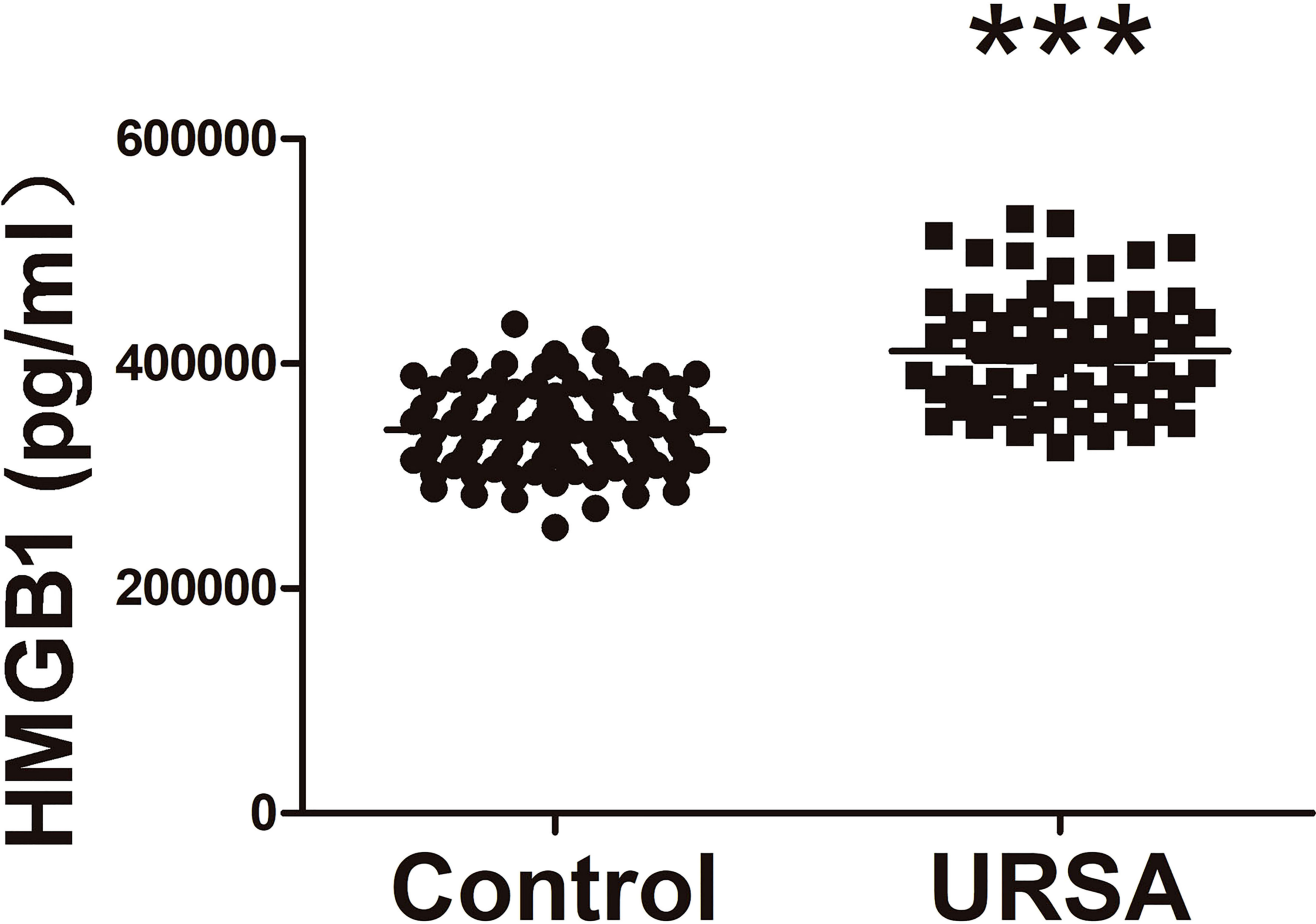
Comparison of plasma HMGB1 concentrations between URSA patients and normal controls. URSA patients (*n* = 60) and normal controls (*n* = 90). ***P < 0.001.

We compared 60 URSA patients who were not treated with aspirin with the control population. As shown in **Figure 2**, HMGB1 levels were lower in peripheral blood of control group (normal person) compared with URSA group. The characteristics of two groups including maternal age, BMI, and previous miscarriages are shown in **Supplementary Tables S1, S2**. The results demonstrated the existence of excessive HMGB1 in URSA maternal circulation. Thus, the next step is to reveal the downregulation process of HMGB1 by drugs.

### Administration of aspirin decreases the plasma concentrations of HMGB1 in URSA patients

**Figure 3:**
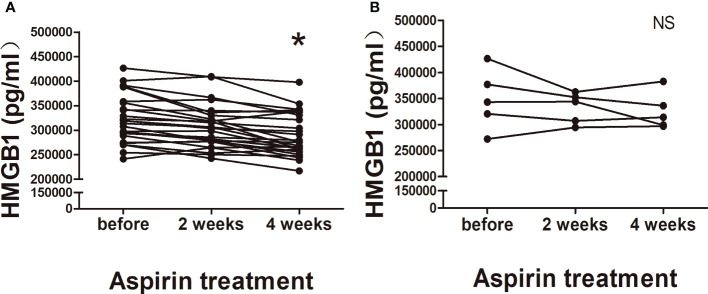
Changes of plasma HMGB1 concentrations in aspirin treatment group. **(A)** Nonpregnant URSA patients (*n* = 26). **(B)** URSA patients in early pregnancy (*n* = 5). *P < 0.05; NS, not significant.

In order to prove the inhibitory effect of aspirin on HMGB1, plasma HMGB1 concentrations were measured in aspirin treatment group. Compared with before aspirin treatment, we found that HMGB1 levels showed a continuously decreasing trend in 2 and 4 weeks after aspirin treatment; the degree of decline was statistically significant in 4 weeks (**Figure 3A**). Considering that five URSA patients were already in early pregnancy at their first visit, we compared them separately. As shown in **Figure 3B**, after treatment with aspirin for 2 and 4 weeks, the plasma HMGB1 concentrations of URSA patients during pregnancy also showed a downward trend, but there was no statistical significance. Taken together, these data suggested that aspirin can reduce the expression of HMGB1 in URSA patients, and its intervention effect may be weakened during pregnancy. The characteristics of aspirin treatment group including maternal age, BMI, and previous miscarriages are shown in **Supplementary Table S3**.

### Plasma HMGB1 concentrations of URSA patients are positively correlated with proinflammatory factor IFN-γ

**Figure 4:**
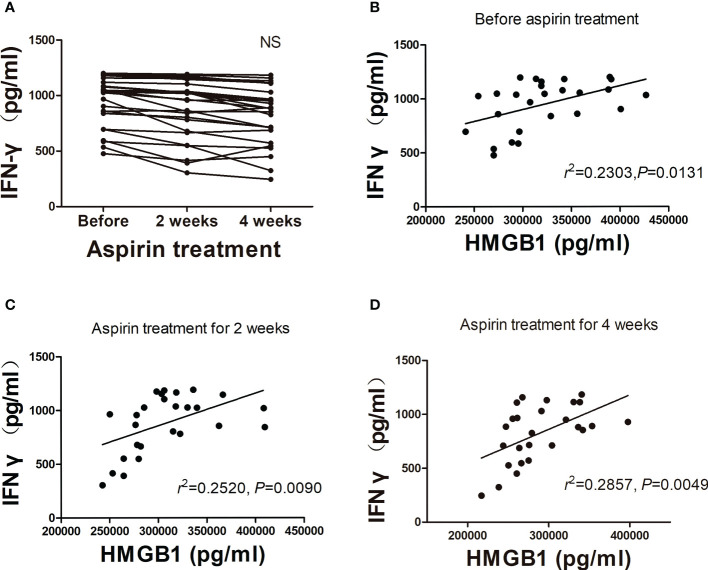
Analysis of plasma IFN-γ concentration in aspirin treatment group. **(A)** Changes of plasma IFN-γ concentrations after treatment with aspirin (*n* = 26). **(B–D)** Correlation analysis of HMGB1 with IFN-γ. NS, not significant.

In order to determine the proinflammatory effect of HMGB1 in URSA patients, we detected the IFN-γ concentrations in aspirin treatment group (nonpregnant women). First, we found that the IFN-γ concentrations also decreased gradually during the three periods of aspirin treatment (**Figure 4A**), especially in 4 weeks after aspirin treatment. Although there was no statistical significance between the three groups, a downward trend was seen after aspirin treatment. Fortunately, no matter before aspirin treatment, 2 weeks or 4 weeks after aspirin treatment, HMGB1 concentrations in the peripheral blood of URSA patients were positively correlated with IFN-γ concentrations (*r* = 0.480, *r* = 0.502, *r* = 0.535, *P* < 0.05; **Figures 4B–D**). The results showed that after the continuous administration of aspirin for 2 and 4 weeks in URSA patients, the levels of HMGB1 and IFN-γ continued to decrease and both of them were correlated. Based on this, we speculate that low-dose aspirin may have an ameliorating effect on HMGB1-induced aseptic inflammation.

### Analysis of the pregnancy and obstetric outcomes in patients of URSA treated with aspirin

We followed up 31 URSA patients who have completed aspirin monitoring treatment. By December 2021, there were 21 deliveries, three of which were assisted reproduction pregnancy and one gave birth to twins. The obstetric outcomes are shown in **Table 1**. Two cases were in pregnancy, and both were more than 12 weeks gestation. Thus, low-dose aspirin ultimately improved pregnancy and obstetric outcomes in URSA patients.

**Table 1:** Obstetric outcomes of URSA patients treated with aspirin.

NO.	Gestational age (weeks)^+days^	Birth date	Birth weight (g)	Body length (cm)	Apgar 10 min	Healthy
1	40	2019.10.07	3800	51	10	Yes
2	37^+1^	2019.10.18	3600	50	10	Yes
3	38^+2^	2019.10.25	3600	50	10	Yes
4	40	2019.10.31	3000	49	10	Yes
5	41	2019.11.03	3720	52	10	Yes
6	34^+5^	2019.12.05	2100	41	9	Yes
7	42^+2^	2020.01.09	4050	52	10	Yes
8	40^+1^	2020.04.24	3600	50	9	Yes
9	38^+6^	2020.05.12	3200	50	10	Yes
10-1	35^+5^(1)	2020.06.10	2450	47	10	Yes
10-2	35^+5^(2)	2020.06.10	2250	46	10	Yes
11	40^+3^	2021.02.06	3570	52	10	Yes
12	40	2021.06.12	2350	50	10	Yes
13	39^+2^	2021.06.22	3500	50	10	Yes
14	41	2021.06.24	3750	51	10	Yes
15	39^+1^	2021.07.03	3150	50	10	Yes
16	40^+4^	2021.07.22	3200	50	10	Yes
17	39^+3^	2021.09.08	2800	47	10	Yes
18	40^+4^	2021.10.22	4000	51	10	Yes
19	39	2021.11.03	2900	50	10	Yes
20	40^+7^	2021.11.22	3700	50	10	Yes
21	39^+3^	2021.12.12	3400	50	10	Yes

## Discussion

In the present study, we discovered that URSA patients had higher HMGB1 levels compared with control women, treatment with low-dose aspirin can effectively reduce the concentrations of HMGB1 in peripheral blood, the concentrations of proinflammatory factor IFN-γ were also decreased, and the degree of their decrease showed a time-dependent manner. Moreover, the expression of HMGB1 in plasma was positively correlated with IFN-γ.

Recurrent miscarriage is a very common pregnancy complication which perplexes both couples of childbearing age and clinician. Moreover, nearly 50% of RSA patients cannot find the exact etiology. A third of women attending specialist clinics are clinically depressed, and one in five has levels of anxiety that are similar to those in psychiatric outpatient populations (19). In recent years, many of researches that focus about the pathogeny and treatment of URSA have turned to maternal-fetal immune microenvironment, which involves cells, cytokines, and some endogenous damage media. HMGB1 is a highly conserved DAMP that plays a critical role in mediating the immune responses to inflammatory injury. Stimulation of neutrophils and monocytes with HMGB1 can induce cytokines secretion and increase the interaction between Mac-1 and RAGE that contributes immune cells to adhere to activated vascular endothelium and migrate into inflammatory tissue (20, 21). In our previous researches, the upregulation of HMGB1 and its receptors levels in the peripheral blood and maternal-fetal interface in URSA patients or mice indicated their involvement in the pathogenesis of URSA (17, 18).

Although multiple therapies effective to block HMGB1 were reported, such as neutralizing antibodies, receptors blocker, and peptides, their safety to pregnant women may need further study. Clinically, low-dose aspirin is widely used in RSA, which main mechanism is to inhibit platelet aggregation and adhesion, improve coagulation function, alleviate prethrombotic state, and block the hypercoagulation response in the decidua space (22). Our study concentrates on the molecular mechanism that aspirin can target HMGB1. As a classic anti-inflammatory drug, we believe that aspirin is safe and effective for URSA patients in downregulating HMGB1. As the results shown (**Figure 3A**), the plasma HMGB1 concentrations decreased in time dependently after aspirin treated, which demonstrated that long-term use of low-dose aspirin can effectively reduce the high levels of HMGB1 in URSA patients.

In recent years, the balance between Th1/Th2/Th17 and Treg cells has been recognized as a new paradigm for successful pregnancy (23). The Th17 and Treg cells immune imbalance may be a crucial immune factor in RSA (24). The declined Treg cell levels might stimulate the failure of implantation and an augmented occurrence of Th17 cells in peripheral blood and deciduas may lead to unexplained recurrent miscarriage (25). The administration of excessive Th1-type cytokine such as IFN-γ can induce abortion in mice (26). In addition, it has been reported that IFN-γ can induce HMGB1 release from macrophages (27). In this study, due to the limitation of research sample size, the decrease of IFN-γ in aspirin-treated patients was not statistically significant. However, positive correlations were found between HMGB1 and IFN-γ, and the plasma IFN-γ concentrations in aspirin treatment group also showed a downward trend. These results suggest that aspirin, as a blocker of HMGB1, can interfere with the production of the proinflammatory factor IFN-γ.

The main limitation of our study was with relatively small sample size. At present, there are many therapeutic interventions for recurrent abortion and fetal protection. Many patients had to be treated with multiple drugs before and after pregnancy, such as low-molecular-weight heparin, progesterone, prednisone, and so on. Because we want to observe the inhibitory effect of aspirin on HMGB1 alone, we must exclude the interference of other drugs, which led many URSA patients to withdraw from the study. In this study, only four patients with assisted reproduction pregnancy were treated with other drugs after aspirin monitoring treatment. We look forward to expanding the sample size in future research and further exploring the inhibitory effect of aspirin on the inflammatory response pathway induced by HMGB1.

In conclusion, we demonstrated the dynamic trend of low-dose aspirin downregulating HMGB1 for the first time, proinflammatory factor IFN-γ is closely related to the secretion of HMGB1, low-dose aspirin may ameliorate pregnancy outcomes of URSA patients by inhibiting the HMGB1-induced inflammatory activation. Moreover, HMGB1 can be used as an important indicator to estimate the risk of recurrent abortion so as to achieve early treatment.

## Data availability statement

The datasets presented in this study can be found in online repositories. The names of the repository/repositories and accession number(s) can be found in the article/**Supplementary Material**.

## Ethics statement

This study was approved by the medical ethics committee of the First Affiliated Hospital of Anhui Medical University and the relevant hospitals (Ethical approval no. PJ2018-02-09, clinical trial registration: ChiCTR1800015403). The patients/participants provided their written informed consent to participate in this study. Written informed consent was obtained from the individual(s) for the publication of any potentially identifiable images or data included in this article.

## Author contributions

XX designed the study and drafted the article. JW collected the samples and drafted the article. HZ revised the article critically for important intellectual content. DZ, JY, JL, XW, WY, QH and YR collected the samples. ZZ analyzed the data. PZ and ZW collected the data. YC give final approval of the version to be published.

## Funding

This research was supported by the National Natural Sciences Foundation of China (No. 32000642), the Natural Science Foundation of Anhui Province (No.1908085MH244), the Nonprofit Central Research Institute Fund of Chinese Academy of Medical Sciences (No. 2019PT310002), the Research Fund of Anhui Institute of translational medicine (ZHYX2020A001 and 2021zhyx-B16).

## Acknowledgments

The authors wish to acknowledge all the staff of the IVF center at the First Affiliated Hospital of Anhui Medical University for their support for this study. We express thanks to Junqiang Zhang, Huiru Chen and Zhen Yu from Anhui Province Key Laboratory of Reproductive Health and Genetics, Anhui Medical University, for experimental instruction.

## Conflict of interest

The authors declare that the research was conducted in the absence of any commercial or financial relationships that could be construed as a potential conflict of interest.

## Publisher’s note

All claims expressed in this article are solely those of the authors and do not necessarily represent those of their affiliated organizations, or those of the publisher, the editors and the reviewers. Any product that may be evaluated in this article, or claim that may be made by its manufacturer, is not guaranteed or endorsed by the publisher.
