# Supplementary material for: Low-dose aspirin protects unexplained recurrent spontaneous abortion via downregulation of HMGB1 inflammation activation
Source: Front Endocrinol (Lausanne). 2022 Nov 17;13:914030. doi: 10.3389/fendo.2022.914030 (PMC9712724; doi:10.3389/fendo.2022.914030)
Supplement: Supplementary file 1 [file Table_1.docx]

**Supplemental Table S 1 Patients Characteristics in URSA group**

| Number | Age | BMI | Pregnancy history | Details |  |
| --- | --- | --- | --- | --- | --- |
| 1 | 26 | 20.8 | 0-0-2-0 | Embryo stopped development | 2 |
| 2 | 25 | 27.5 | 0-0-2-0 | Embryo stopped development | 2 |
| 3 | 23 | 26.7 | 0-0-2-0 | Spontaneous abortion | 2 |
| 4 | 34 | 27.1 | 1-0-2-1 | Spontaneous delivery  Embryo stopped development | 1  2 |
| 5 | 27 | 21.5 | 0-0-2-0 | Spontaneous abortion  Embryo stopped development | 1  1 |
| 6 | 26 | 24.1 | 0-0-5-0 | Induced abortion  (unplanned pregnancy)  Spontaneous abortion | 3  2 |
| 7 | 30 | 19.5 | 0-0-3-0 | Induced abortion  (unplanned pregnancy)  Embryo stopped development  Spontaneous abortion | 1  1  1 |
| 8 | 29 | 21.2 | 0-0-2-0 | Embryo stopped development | 2 |
| 9 | 33 | 22.0 | 1-0-2-1 | Spontaneous delivery  Spontaneous abortion | 1  2 |
| 10 | 29 | 21.5 | 0-0-3-0 | Induced abortion  (unplanned pregnancy)  Embryo stopped development | 1  2 |
| 11 | 28 | 23.0 | 1-0-2-1 | Spontaneous delivery  Embryo stopped development | 1  2 |
| 12 | 27 | 21.5 | 0-0-3-0 | Induced abortion  (unplanned pregnancy)  Embryo stopped development  Spontaneous abortion | 1  1  1 |
| 13 | 29 | 23.9 | 0-0-2-0 | Embryo stopped development | 2 |
| 14 | 30 | 21.0 | 0-0-2-0 | Embryo stopped development | 2 |
| 15 | 32 | 26.9 | 1-0-2-1 | Cesarean delivery  Spontaneous abortion | 1  2 |
| 16 | 33 | 19.8 | 0-0-2-0 | Spontaneous abortion  Embryo stopped development | 1  1 |
| 17 | 34 | 23.1 | 1-0-3-1 | Induced abortion  (unplanned pregnancy)  Spontaneous delivery  Embryo stopped development | 1  1  2 |
| 18 | 37 | 19.2 | 1-0-3-1 | Induced abortion  (unplanned pregnancy)  Spontaneous delivery  Embryo stopped development | 2  1  2 |
| 19 | 32 | 25.9 | 1-0-2-1 | Spontaneous delivery  Embryo stopped development | 1  2 |
| 20 | 30 | 19.5 | 1-0-2-1 | Spontaneous delivery  Embryo stopped development | 1  2 |
| 21 | 32 | 18.4 | 1-0-3-1 | Spontaneous delivery  Inevitable abortion  Embryo stopped development | 1  1  2 |
| 22 | 31 | 18.6 | 0-0-2-0 | Spontaneous abortion  Embryo stopped development | 1  1 |
| 23 | 32 | 22.1 | 0-0-2-0 | Spontaneous abortion  Embryo stopped development | 1  1 |
| 24 | 20 | 24.4 | 0-0-2-0 | Embryo stopped development | 2 |
| 25 | 34 | 18.0 | 0-0-2-0 | Spontaneous abortion  Embryo stopped development | 1  1 |
| 26 | 36 | 24.7 | 2-0-2-2 | Spontaneous delivery  Embryo stopped development | 2  2 |
| 27 | 28 | 19.0 | 0-0-2-0 | Embryo stopped development | 2 |
| 28 | 28 | 23.6 | 0-0-2-0 | Embryo stopped development | 2 |
| 29 | 31 | 21.3 | 1-0-2-1 | Spontaneous delivery  Embryo stopped development | 1  2 |
| 30 | 30 | 17.4 | 0-0-2-0 | Embryo stopped development | 2 |
| 31 | 38 | 24.6 | 0-0-2-0 | Embryo stopped development  Spontaneous abortion | 1  1 |
| 32 | 24 | 27.4 | 0-0-2-0 | Embryo stopped development | 2 |
| 33 | 37 | 23.1 | 1-0-4-1 | Cesarean delivery  Induced abortion  (unplanned pregnancy)  Embryo stopped development | 1  1  3 |
| 34 | 31 | 24.1 | 0-0-3-0 | Embryo stopped development  Induced abortion  (unplanned pregnancy) | 2  1 |
| 35 | 24 | 22.0 | 0-0-2-0 | Embryo stopped development | 2 |
| 36 | 27 | 25.7 | 0-0-2-0 | Embryo stopped development | 2 |
| 37 | 23 | 23.0 | 0-0-2-0 | Embryo stopped development | 2 |
| 38 | 24 | 18.8 | 0-0-2-0 | Embryo stopped development | 2 |
| 39 | 25 | 19.5 | 0-0-2-0 | Embryo stopped development | 2 |
| 40 | 29 | 21.5 | 0-0-3-0 | Embryo stopped development | 3 |
| 41 | 32 | 18.6 | 0-0-2-0 | Embryo stopped development  Spontaneous abortion | 1  1 |
| 42 | 34 | 30.5 | 0-0-6-0 | Spontaneous abortion  Embryo stopped development | 1  5 |
| 43 | 38 | 24.8 | 1-0-6-1 | Spontaneous delivery  Medical abortion  Induced abortion  (unplanned pregnancy)  Inevitable abortion  Embryo stopped development | 1  1  1  2  2 |
| 44 | 38 | 23.2 | 2-0-3-2 | Spontaneous delivery  Cesarean delivery  Medical abortion  (unplanned pregnancy)  Embryo stopped development | 1  1  1  2 |
| 45 | 32 | 25.4 | 2-0-2-2 | Spontaneous delivery  Spontaneous abortion  Embryo stopped development | 2  1  1 |
| 46 | 30 | 22.0 | 0-0-3-0 | Embryo stopped development | 3 |
| 47 | 23 | 18.7 | 0-0-2-0 | Embryo stopped development | 2 |
| 48 | 36 | 28.7 | 1-0-3-1 | Cesarean delivery  Embryo stopped development | 1  3 |
| 49 | 34 | 22.6 | 0-0-2-0 | Embryo stopped development | 2 |
| 50 | 30 | 20.7 | 0-0-2-0 | Embryo stopped development | 2 |
| 51 | 25 | 20.4 | 0-0-2-0 | Spontaneous abortion  Embryo stopped development | 1  1 |
| 52 | 37 | 19.1 | 0-0-2-0 | Embryo stopped development  Spontaneous abortion | 1  1 |
| 53 | 33 | 26.0 | 0-0-3-0 | Spontaneous abortion  Embryo stopped development | 1  2 |
| 54 | 27 | 23.0 | 0-0-2-0 | Embryo stopped development | 2 |
| 55 | 34 | 19.9 | 0-0-3-0 | Induced abortion  Embryo stopped development | 1  2 |
| 56 | 29 | 22.0 | 0-0-2-0 | Spontaneous abortion | 2 |
| 57 | 25 | 23.3 | 0-0-2-0 | Embryo stopped development | 2 |
| 58 | 35 | 28.0 | 1-0-2-1 | Spontaneous delivery  Embryo stopped development | 1  2 |
| 59 | 30 | 24.0 | 0-0-4-0 | Induced abortion  (unplanned pregnancy)  Embryo stopped development | 2  2 |
| 60 | 26 | 23.8 | 0-0-2-0 | Embryo stopped development | 2 |
