# Supplementary material for: Low-dose aspirin protects unexplained recurrent spontaneous abortion via downregulation of HMGB1 inflammation activation
Source: Front Endocrinol (Lausanne). 2022 Nov 17;13:914030. doi: 10.3389/fendo.2022.914030 (PMC9712724; doi:10.3389/fendo.2022.914030)
Supplement: Supplementary file 2 [file Table_2.docx]

**Supplemental Table S2 Patients Characteristics in Control group**

| Number | Age | BMI | Pregnancy history | Details |  |
| --- | --- | --- | --- | --- | --- |
| 1 | 29 | 19.5 | 1-0-0-1 | Spontaneous delivery | 1 |
| 2 | 29 | 20.4 | 1-0-0-1 | Spontaneous delivery | 1 |
| 3 | 29 | 27.1 | 1-0-0-1 | Cesarean delivery | 1 |
| 4 | 29 | 28.2 | 0-0-1-0 | Induced abortion  (unplanned pregnancy) | 1 |
| 5 | 31 | 22.9 | 1-0-0-1 | Spontaneous delivery | 1 |
| 6 | 22 | 21.3 | 0-0-0-0 | / |  |
| 7 | 29 | 19.6 | 1-0-0-1 | Spontaneous delivery | 1 |
| 8 | 28 | 24.1 | 1-0-0-1 | Spontaneous delivery | 1 |
| 9  10 | 27  30 | 23.8  23.5 | 0-0-1-0  1-0-0-1 | Induced abortion  (unplanned pregnancy)  Spontaneous delivery | 1  1 |
| 11 | 27 | 22.5 | 1-0-0-1 | Cesarean delivery | 1 |
| 12 | 28 | 23.2 | 1-0-0-1 | Spontaneous delivery | 1 |
| 13 | 27 | 18.8 | 0-0-0-0 | / |  |
| 14 | 29 | 19.1 | 1-0-0-1 | Spontaneous delivery | 1 |
| 15 | 27 | 18.8 | 1-0-0-1 | Spontaneous delivery | 1 |
| 16 | 25 | 23.9 | 0-0-0-0 | / |  |
| 17 | 25 | 23.0 | 1-0-0-1 | Cesarean delivery | 1 |
| 18 | 33 | 22.0 | 1-0-0-1 | Spontaneous delivery | 1 |
| 19 | 25 | 22.2 | 0-0-0-0 | / |  |
| 20 | 27 | 24.0 | 0-0-0-0 | / |  |
| 21 | 29 | 21.5 | 0-0-1-0 | Induced abortion  (unplanned pregnancy) | 1 |
| 22 | 30 | 22.5 | 1-0-0-1 | Spontaneous delivery | 1 |
| 23 | 33 | 24.4 | 1-0-0-1 | Spontaneous delivery | 1 |
| 24 | 33 | 20.7 | 1-0-1-1 | Induced abortion  (unplanned pregnancy)  Spontaneous delivery | 1  1 |
| 25 | 25 | 22.1 | 0-0-0-0 | / |  |
| 26 | 35 | 23.2 | 2-0-0-2 | Spontaneous delivery | 2 |
| 27 | 28 | 25.4 | 0-0-0-0 | / |  |
| 28 | 34 | 19.6 | 1-0-0-1 | Cesarean delivery | 1 |
| 29 | 24 | 30.8 | 0-0-0-0 | / |  |
| 30 | 25 | 22.1 | 0-0-0-0 | / |  |
| 31 | 35 | 20.0 | 1-0-0-1 | Cesarean delivery | 1 |
| 32 | 27 | 22.3 | 0-0-0-0 | / |  |
| 33 | 28 | 27.0 | 0-0-2-0 | Induced abortion  (unplanned pregnancy) | 2 |
| 34 | 30 | 20.5 | 1-0-0-1 | Cesarean delivery | 1 |
| 35 | 30 | 23.8 | 1-0-0-1 | Spontaneous delivery | 1 |
| 36 | 27 | 20.8 | 0-0-0-0 | / |  |
| 37 | 34 | 22.3 | 1-0-0-1 | Spontaneous delivery | 1 |
| 38 | 33 | 23.2 | 1-0-0-1 | Cesarean delivery | 1 |
| 39 | 29 | 22.9 | 0-0-0-0 | / |  |
| 40 | 33 | 18.6 | 1-0-1-1 | Induced abortion  (unplanned pregnancy)  Spontaneous delivery | 1  1 |
| 41 | 25 | 20.5 | 1-0-0-1 | Spontaneous delivery | 1 |
| 42 | 25 | 21.2 | 0-0-0-0 | / |  |
| 43 | 24 | 23.5 | 0-0-0-0 | / |  |
| 44 | 26 | 19.5 | 1-0-0-1 | Spontaneous delivery | 1 |
| 45 | 26 | 20.1 | 0-0-0-0 | / |  |
| 46 | 25 | 20.2 | 1-0-0-1 | Cesarean delivery | 1 |
| 47 | 35 | 28.6 | 2-0-0-2 | Spontaneous delivery | 2 |
| 48 | 31 | 25.1 | 1-0-0-1 | Spontaneous delivery | 1 |
| 49 | 34 | 18.7 | 0-0-2-0 | Induced abortion  (unplanned pregnancy) | 2 |
| 50 | 35 | 21.1 | 1-0-0-1 | Spontaneous delivery | 1 |
| 51 | 26 | 17.8 | 0-0-0-0 | / |  |
| 52 | 28 | 20.4 | 1-0-0-1 | Spontaneous delivery | 1 |
| 53 | 30 | 23.4 | 0-0-1-0 | Induced abortion  (unplanned pregnancy) | 1 |
| 54 | 26 | 20.8 | 0-0-0-0 | / |  |
| 55 | 26 | 28.1 | 0-0-0-0 | / |  |
| 56 | 30 | 24.2 | 1-0-0-1 | Spontaneous delivery | 1 |
| 57 | 31 | 26.0 | 1-0-0-1 | Spontaneous delivery | 1 |
| 58 | 27 | 20.3 | 1-0-0-1 | Cesarean delivery | 1 |
| 59 | 29 | 19.8 | 1-0-0-1 | Spontaneous delivery | 1 |
| 60 | 26 | 20.4 | 0-0-0-0 | / |  |
| 61 | 27 | 20.8 | 1-0-0-1 | Spontaneous delivery | 1 |
| 62 | 24 | 23.0 | 0-0-0-0 | / |  |
| 63 | 34 | 18.0 | 1-0-0-1 | Spontaneous delivery |  |
| 64 | 32 | 30.5 | 1-0-0-1 | Cesarean delivery | 1 |
| 65 | 29 | 22.7 | 1-0-0-1 | Spontaneous delivery | 1 |
| 66 | 27 | 25.6 | 0-0-0-0 | / |  |
| 67 | 29 | 30.5 | 1-0-0-1 | Spontaneous delivery | 1 |
| 68 | 27 | 21.8 | 0-0-0-0 | / |  |
| 69 | 30 | 20.9 | 1-0-1-1 | Spontaneous delivery  Induced abortion  (unplanned pregnancy) | 1  1 |
| 70 | 29 | 18.4 | 0-0-0-0 | / |  |
| 71 | 30 | 25.4 | 1-0-0-1 | Spontaneous delivery | 1 |
| 72 | 31 | 27.5 | 1-0-0-1 | Spontaneous delivery | 1 |
| 73 | 35 | 19.5 | 2-0-0-2 | Spontaneous delivery | 2 |
| 74 | 27 | 19.1 | 1-0-0-1 | Spontaneous delivery | 1 |
| 75 | 28 | 29.3 | 1-0-1-1 | Induced abortion  (unplanned pregnancy)  Cesarean delivery | 1  1 |
| 76 | 28 | 21.9 | 1-0-0-1 | Spontaneous delivery | 1 |
| 77 | 27 | 23.9 | 1-0-0-1 | Spontaneous delivery | 1 |
| 78 | 26 | 18.4 | 0-0-0-0 | Spontaneous delivery | 1 |
| 79 | 27 | 17.2 | 1-0-0-1 | Cesarean delivery | 1 |
| 80 | 24 | 23.9 | 0-0-0-0 | / |  |
| 81 | 27 | 23.6 | 1-0-0-1 | Spontaneous delivery | 1 |
| 82 | 31 | 22.0 | 0-0-0-0 | Spontaneous delivery | 1 |
| 83 | 27 | 21.0 | 1-0-0-1 | Spontaneous delivery | 1 |
| 84 | 26 | 25.1 | 0-0-0-0 | / |  |
| 85 | 26 | 22.5 | 0-0-0-0 | / |  |
| 86 | 28 | 19.2 | 1-0-0-1 | Spontaneous delivery | 1 |
| 87 | 33 | 21.1 | 1-0-1-1 | Spontaneous delivery  Induced abortion  (unplanned pregnancy) | 1  1 |
| 88 | 26 | 23.4 | 1-0-0-1 | Spontaneous delivery | 1 |
| 89 | 27 | 20.9 | 1-0-0-1 | Spontaneous delivery | 1 |
| 90 | 30 | 22.1 | 1-0-0-1 | Cesarean delivery | 1 |
