# Supplementary material for: Low-dose aspirin protects unexplained recurrent spontaneous abortion via downregulation of HMGB1 inflammation activation
Source: Front Endocrinol (Lausanne). 2022 Nov 17;13:914030. doi: 10.3389/fendo.2022.914030 (PMC9712724; doi:10.3389/fendo.2022.914030)
Supplement: Supplementary file 3 [file Table_3.docx]

**Supplemental Table S3 Patients characteristics in aspirin treatment group**

| Number | Age | BMI | Pregnancy history | Details |  |
| --- | --- | --- | --- | --- | --- |
| 1 | 30 | 19.8 | 0-0-2-0 | Embryo stopped development | 2 |
| 2 | 30 | 23.4 | 0-1-3-1 | Embryo stopped development  Spontaneous abortion  Preterm labor | 2  1  1 |
| 3 | 26 | 21.5 | 0-0-2-0 | Embryo stopped development  Spontaneous abortion | 1  1 |
| 4 | 26 | 17.6 | 0-0-2-0 | Embryo stopped development  Spontaneous abortion | 1  1 |
| 5 | 28 | 19.6 | 1-0-2-1 | Spontaneous delivery  Embryo stopped development | 1  2 |
| 6 | 23 | 23.4 | 1-0-2-1 | Cesarean delivery  Spontaneous abortion  Embryo stopped development | 1  1  1 |
| 7 | 32 | 17.3 | 0-0-2-0 | Spontaneous abortion  Embryo stopped development | 1  1 |
| 8 | 34 | 25.0 | 1-0-3-1 | Embryo stopped development  Spontaneous delivery | 3  1 |
| 9 | 30 | 21.5 | 0-0-2-0 | Spontaneous abortion | 2 |
| 10 | 30 | 24.4 | 0-0-2-0 | Embryo stopped development  Spontaneous abortion | 1  1 |
| 11 | 31 | 20.3 | 0-0-2-0 | Embryo stopped development  Spontaneous abortion | 1  1 |
| 12 | 30 | 24.7 | 0-0-2-0 | Embryo stopped development | 2 |
| 13 | 27 | 21.2 | 0-0-2-0 | Spontaneous abortion  Embryo stopped development | 1  1 |
| 14 | 26 | 29.1 | 0-0-2-0 | Spontaneous abortion  Embryo stopped development | 1  1 |
| 15 | 31 | 18.1 | 0-0-2-0 | Embryo stopped development | 2 |
| 16 | 33 | 20.1 | 0-0-2-0 | Embryo stopped development  Spontaneous abortion | 1  1 |
| 17 | 20 | 19.6 | 0-0-2-0 | Embryo stopped development | 2 |
| 18 | 29 | 23.9 | 0-0-3-0 | Embryo stopped development | 3 |
| 19 | 29 | 24.1 | 0-0-2-0 | Embryo stopped development | 2 |
| 20 | 31 | 22.5 | 1-1-2-2 | Spontaneous delivery  Preterm labor  Spontaneous abortion | 1  1  2 |
| 21 | 27 | 22.1 | 0-0-2-0 | Embryo stopped development | 2 |
| 22 | 30 | 21.0 | 1-0-2-1 | Cesarean delivery  Embryo stopped development | 1  2 |
| 23 | 31 | 22.2 | 0-0-2-0 | Embryo stopped development | 2 |
| 24 | 31 | 29.6 | 1-0-2-1 | Spontaneous delivery  Embryo stopped development | 1  2 |
| 25 | 32 | 24.8 | 0-0-6-0 | Spontaneous abortion  Embryo stopped development | 1  5 |
| 26 | 29 | 24.9 | 0-0-2-0 | Embryo stopped development | 2 |
| 27 | 29 | 23.6 | 0-0-4-0 | Spontaneous abortion | 4 |
| 28 | 24 | 22.0 | 0-0-2-0 | Spontaneous abortion  Embryo stopped development | 1  1 |
| 29 | 26 | 24.4 | 0-0-2-0 | Embryo stopped development | 2 |
| 30 | 37 | 20.8 | 1-0-3-1 | Spontaneous delivery  Embryo stopped development | 1  3 |
| 31 | 28 | 20.7 | 0-0-2-0 | Embryo stopped development | 2 |
